# New York State Dental Society

**Published:** 1869-02

**Authors:** 


### NEW YORK STATE DENTAL SOCIETY.

An adjourned meeting of this Society was held at Albany, February 2d, 1869.

The organization was fully perfected by the adoption of by-laws and a code of ethics.

The President read an inaugural address, giving a concise history of the rise and progress of Dentistry in the United States, showing the rapid advancement made during the nineteenth century, and that from the absence of proper restrictions many were entering upon its practice, without going through a proper course of education, and that the ranks of the Dental profession were now swollen to such magnitude that the time had evidently arrived when some legal barrier should be raised and a higher degree of qualification demanded for the protection of the public and the salvation of such important organs as the teeth. The State Legislature, on the 7th day of April, 1868, passed a law to regulate the practice of Dentistry, and for the organization of Dental Societies, which will prove of great benefit, although inadequate to bring about a full reform. It is certain that any body of professional or scientific men are better judges of the qualifications of those engaged in their particular calling than the public at large, and that each profession, especially in those branches of science pertaining to health, should be left to govern its own members.

Dr. John Allen, of New York, read a paper on artificial dentures, which evinced great research and was eminently practical, showing what could be accomplished in the perfect restoration of the contour of the face and the natural expression, the loss of which necessarily results from the loss of the teeth.

Prof. N. W. Kingsley, of the New York College of Dentistry, read a short paper on Dental art, introductory to an extempore lecture, illustrated by a series of crayon sketches on canvas, and also demonstrated in one case on the human subject, showing what wonderful changes may take place in the human face divine by the loss or restoration of the teeth. The doctor struck out in an entirely new field in physiology, and evinced himself the true artist that he is.

The State Medical Society being in session at this time, sent in a resolution to the Dental Society, expressing their cordial approbation and countenance in the steps taken for the elevation and advancement of Dental and Medical science.

It may not be generally known that, in conformity to the above named Dental law, a district society has been formed in each of the eight judicial districts of the State, and also the State Dental Society, which is composed of eight delegates from each of the district societies, permanent members elected by the State Society from Dentists in this State, and honorary members from other States or countries.

OFFICERS.

President—Dr. A. Westcott, Syracuse.
Vice President—Dr. W. B. Hurd, Brooklyn.
Secretary—Dr. L. W. Rogers, Utica.
Treasurer—Dr. B. T. Whitney, Buffalo.

The Board of Censors is composed of

1st District—Dr. J. G. Ambler, New York.
2d District—Dr. W. B. Hurd, Brooklyn.
3d District—Dr. Alex. Nelson, Albany.
4th District—Dr. Z. Cotton, Cambridge.
5th District—Dr. A. Westcott, Syracuse.
6th District—Dr. R. Walker, Oswego.
7th District—Dr. F. French, Rochester.
8th District—Dr. R. G. Snow, Buffalo.

A full list of Delegates was appointed to the American Dental Association, which is to meet at Saratogo on the first Tuesday of August. Also Dr. J. G. Ambler, of New York, as delegate to the Dental Society of the State of Pennsylvania; Dr. George, E. Hayes, of Buffalo, delegate to the Ohio State Dental Society, and Dr. B. T. Whitney, of Buffalo, delegate to the Dental Association of Ontario, Canada.

It is now very important that every Dentist should connect himself with his district society, and thus bring himself within the pale of the law, and also make application to the Board of Censors of the State Society for a diploma, or to the censors of his district society for a license. The annual meeting will be held on the last Tuesday in July, 1869.

